# Application of metagenomic next-generation sequencing in the diagnosis of pulmonary invasive fungal disease

**DOI:** 10.3389/fcimb.2022.949505

**Published:** 2022-09-27

**Authors:** Chengtan Wang, Zhiqing You, Juanjuan Fu, Shuai Chen, Di Bai, Hui Zhao, Pingping Song, Xiuqin Jia, Xiaoju Yuan, Wenbin Xu, Qigang Zhao, Feng Pang

**Affiliations:** ^1^ Department of Clinical Laboratory, Liaocheng People’s Hospital, Liaocheng, China; ^2^ Department of Clinical Laboratory, Liaocheng Third People’s Hospital, Liaocheng, China; ^3^ Department of Virology, School of Public Health, Shandong University, Jinan, China; ^4^ The Key Laboratory of Molecular Pharmacology, Liaocheng People’s Hospital, Liaocheng, China; ^5^ Department of Gastroenterology, Liaocheng People’s Hospital, Liaocheng, China

**Keywords:** clinical mNGS, invasive fungal infections, immunosuppressed patients, pneumonia, pathogen detection

## Abstract

**Background:**

Metagenomic next-generation sequencing (mNGS) is increasingly being used to detect pathogens directly from clinical specimens. However, the optimal application of mNGS and subsequent result interpretation can be challenging. In addition, studies reporting the use of mNGS for the diagnosis of invasive fungal infections (IFIs) are rare.

**Objective:**

We critically evaluated the performance of mNGS in the diagnosis of pulmonary IFIs, by conducting a multicenter retrospective analysis. The methodological strengths of mNGS were recognized, and diagnostic cutoffs were determined.

**Methods:**

A total of 310 patients with suspected pulmonary IFIs were included in this study. Conventional microbiological tests (CMTs) and mNGS were performed in parallel on the same set of samples. Receiver operating characteristic (ROC) curves were used to evaluate the performance of the logarithm of reads per kilobase per million mapped reads [lg(RPKM)], and read counts were used to predict true-positive pathogens.

**Result:**

The majority of the selected patients (86.5%) were immunocompromised. Twenty species of fungi were detected by mNGS, which was more than was achieved with standard culture methods. Peripheral blood lymphocyte and monocyte counts, as well as serum albumin levels, were significantly negatively correlated with fungal infection. In contrast, C-reactive protein and procalcitonin levels showed a significant positive correlation with fungal infection. ROC curves showed that mNGS [and especially lg(RPKM)] was superior to CMTs in its diagnostic performance. The area under the ROC curve value obtained for lg(RPKM) in the bronchoalveolar lavage fluid of patients with suspected pulmonary IFIs, used to predict true-positive pathogens, was 0.967, and the cutoff value calculated from the Youden index was −5.44.

**Conclusions:**

In this study, we have evaluated the performance of mNGS-specific indicators that can identify pathogens in patients with IFIs more accurately and rapidly than CMTs, which will have important clinical implications.

## Introduction

An increasing number of people are living with immunodeficiencies as a result of medical interventions such as aggressive of prolonged cancer treatments, allogeneic organ and hematopoietic cell transplantation, or the use of corticosteroids for the treatment of autoimmune and autoinflammatory diseases (Ferrarese et al., 2020). The immunosuppressed individual is at a higher risk of developing more invasive fungal infections (IFIs) (Pasqualotto et al., 2006; Ben et al., 2008; Kim et al., 2017; Prattes et al., 2021; Suleyman and Alangaden, 2021), which affects two million individuals each year worldwide (D’enfert and Bougnoux, 2014; Suleyman et al., 2022). The clinical manifestations of IFIs are not typical, and thus, diagnosis mainly relies on etiological evidence. A timely and accurate diagnosis is crucial for the prognosis and survival of patients with IFIs (El-Baba et al., 2020). However, existing techniques such as tissue staining, *in vitro* culture, serological testing, and diagnostic imaging are inadequate for the reliable detection of IFI-causing pathogens. Nearly 60% of IFIs still have an unknown etiology, which delays targeted drug treatment and, in turn, affects patient recovery (Donnelly and Maertens, 2013). The gold standard for diagnosing IFIs is the presence of molds or yeasts in a deep tissue biopsy or a culture obtained by a sterile procedure. Unfortunately, histopathological testing is rarely available in a timely manner because of the risks involved in performing biopsies, whereas culture methods are insensitive and time-consuming. Recently, a real-time PCR approach has shown promise in the detection of *Aspergillus*, *Pneumocystis*, and *Candida* spp. (Donnelly and Maertens, 2013; Donnelly et al., 2020). Despite this, PCR is a hypothesis-driven method that is designed to detect specific pathogens and is therefore not capable of detecting rare and emerging infectious agents (Schlaberg et al., 2017).

Advances in genome sequencing technologies and bioinformatics approaches provide powerful alternatives to overcome such clinical diagnostic challenges (Wang et al., 2021). Metagenomic next-generation sequencing (mNGS) is a culture-independent, hypothesis-independent, broad-spectrum sequencing method, which capable of overcoming the limitations of current diagnostic tests. mNGS enables the universal pathogen detection of viruses, bacteria, fungi, and parasites in a single run (Miao et al., 2018). Since its first application in a clinical setting, mNGS has played an increasingly important role in pathogen identification (Palacios et al., 2008). However, there are still many challenges preventing the routine use of mNGS in the clinic. The primary challenge is that there is currently no common standard for interpreting mNGS results. Although numerous studies have reported the clinical application of mNGS, the majority involve summary retrospective analyses of the diagnostic performance of this novel methodology on a heterogeneous group of patients (Qian et al., 2020; Gu et al., 2021; Liu et al., 2022a). mNGS analyses focusing on the diagnosis of IFIs are rare. Furthermore, it has not yet been reported which indicator is most suitable for distinguishing between pathogens and colonizing/contaminant microorganisms that are present in the sample, the reagents, or the laboratory environment.

In this study, we evaluated the performance of an mNGS indicator [lg(RPKM)] to identify pathogenic fungi in patients with suspected pulmonary IFIs and established a cutoff value for the identification of pathogens. Using this cutoff value, we compared the diagnostic value of mNGS with conventional microbiological tests (CMTs). Finally, we analyzed the correlation between eight clinical infection indicators and various forms of fungal infection.

## Materials and methods

### Study setting and design

**Figure 1:**
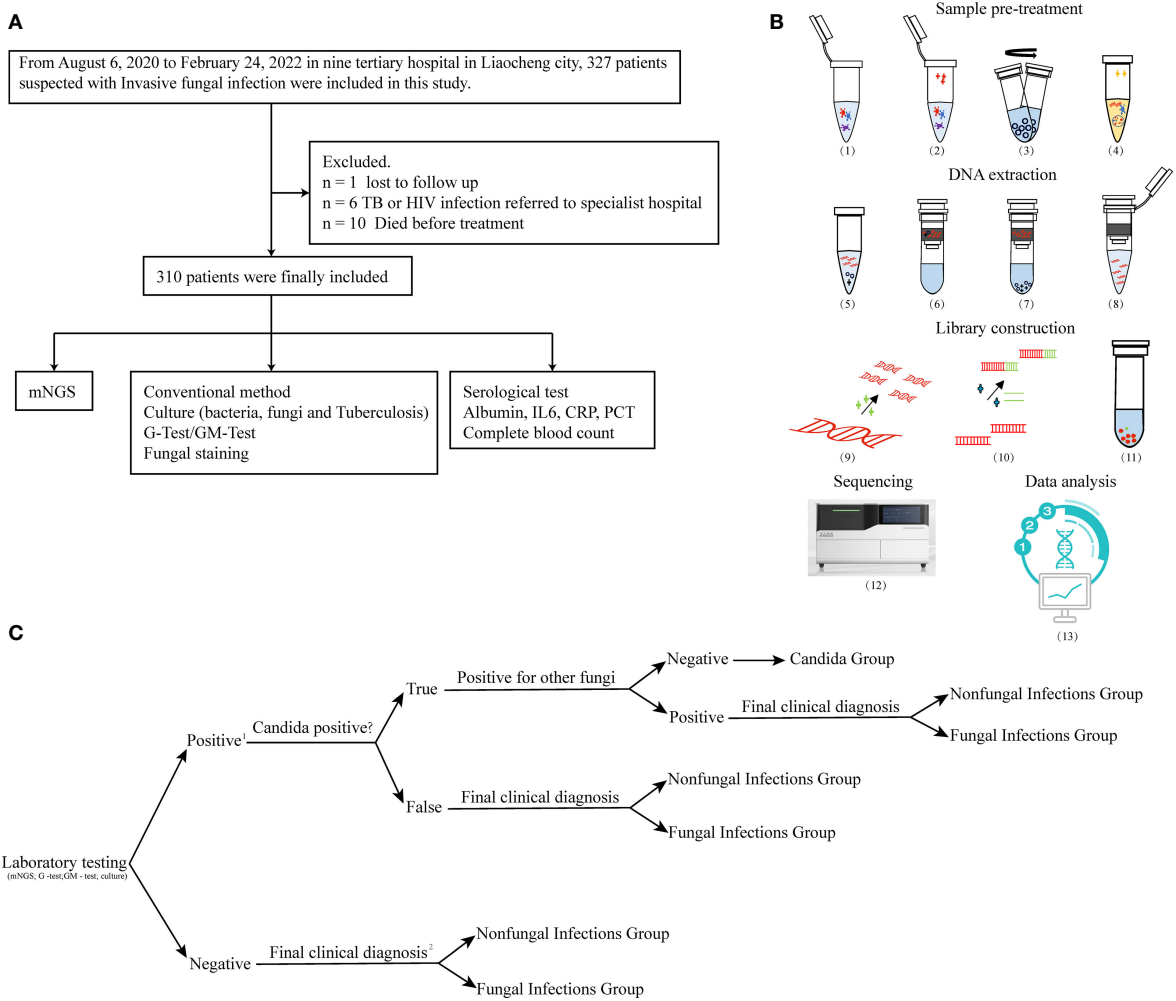
Flowchart. **(A)** Flowchart of participant enrollment criteria. **(B)** Workflow of mNGS. Sample pre-treatment includes the following: (1) inactivation: dry bath at 56°C for 30 min; (2) lysozyme treatment at 30°C for 10 min; (3) add micro glass grinding beads to the lysate and vortex the samples at 1,000 x g for 20 min. (4) Lysis: collect supernatants and add proteinase k and lysis solution at 70°C for 15 min. DNA extraction includes (5) application of 100% ethanol concentration to induce nucleic acid precipitation, (6) DNA binding, (7) washing, and (8) elution. Library construction includes (9) nucleic acid fragmentation (10), adapter ligation, (11) DNB library preparation, (12) sequencing in BGI-seq 50, and (13) data analysis. **(C)** Group analysis workflow for the samples included in the study. 1. A positive result was defined as the presence of fungal infection that was detected by any of the following methods: mNGS, G and GM tests, and culture. 2. The final clinical diagnosis of IFIs was made by a decision-making group comprised of clinical experts, laboratory scientists, and bioinformatics experts, and based on the comprehensive evaluation of all available clinical data. Decision was made according to the revised diagnostic criteria of IFIs outlined by the EORTC/MSG.

We retrospectively reviewed 327 cases of suspected pulmonary IFIs at nine tertiary hospitals in the Shandong Province, China (Liaocheng People’s Hospital, Liaocheng Infectious Diseases Hospital, Liaocheng Traditional Chinese Medicine Hospital, Liaocheng Third People’s Hospital, Liaocheng Brain Hospital, Liaocheng Dongchangfu District Hospital, Dong’e County People’s Hospital, Guan County Central Hospital, and Guan County Traditional Chinese Medicine Hospital) between August 2020 and February 2022. We followed the revised diagnostic criteria of IFIs outlined by the European Organization for Research and Treatment of Cancer/Invasive Fungal Infections Cooperative Group and the National Institute of Allergy and Infectious Diseases Mycoses Study Group (EORTC/MSG) (De Pauw et al., 2008; Alexander et al., 2021). See **supplementary document Table S1** for our diagnostic criteria. Eventually, 310 patients with suspected pulmonary IFIs were enrolled and 17 were excluded (**Figure 1A**). Clinical data of patients were rigorously evaluated, summarized, and compiled until death or hospital discharge. All the enrolled patients were subjected to mNGS, CMTs, and a serological test (**Figure 1A**). We divided the 310 subjects into three groups: 1) the fungal infections group, 2) the non-fungal infections group, and 3) the *Candida* group according to the grouping principles outlined in **Figure 1C**. This study was approved by the Medical Ethics Committee of Liaocheng People’s Hospital (No. 2022097). All samples in this study were anonymized, and the experiments were performed in accordance with the Declaration of Helsinki.

### Conventional microbiological testing and examination of infection indices

Bronchoalveolar lavage fluid (BALF) samples collected from patients with suspected pulmonary IFIs underwent CMTs and mNGS in parallel. CMTs were carried out according to the standard procedures (Donnelly et al., 2020). The 1,3-β-D glucan (G) assay (the G test) and galactomannan (GM) assay (the GM test) were performed according to the manufacturer’s instructions (Xinuo Biopharmaceutical Co., Ltd., Tianjin, China). The following parameters were subsequently assessed: white blood cell count, neutrophil count, lymphocyte count, monocyte count (Sysmex Co., Ltd., Kobe, Japan), C-reactive protein (CRP) and procalcitonin (PCT) (Shanghai i-Reader Biotech Co., Ltd., Shanghai, China), interleukin-6 (IL-6) [Abbott Diagnostic (Shanghai) Co., Ltd., Shanghai, China], and serum albumin (Beckman Co., Ltd., Brea, America) using the manufacturer’s recommendations.

### Metagenomic next-generation sequencing

The mNGS uses BGI’s high-throughput genetic detection of pathogenic microorganisms (PMseq) process. BALF samples from patients with suspected pulmonary IFIs were collected and stored on dry ice until mNGS. The samples were inactivated at 56°C for 30 min using a dry bath, prior to the addition of wall-lysing enzyme and incubation at 30°C for 10 min. Next, micro glass grinding beads (MGI Tech Co., Ltd., Wuhan, China) were added to the lysate, and the samples were vortexed at 1,000 x g for 20 min. Supernatants were subsequently collected and subjected to DNA extraction using the TIANamp Micro DNA kit (DP316, TIANGEN Biotech, Jiangsu, China). Nucleic acid concentration was determined using the Qubit dsDNA HS Assay kit together with the Qubit 4 Fluorometer (Invitrogen Singapore Pte, Ltd., Singapore, Singapore). Nucleic acid fragmentation (into 250- to 350-bp fragments) was then performed using an enzyme digestion kit (MGI Tech Co., Ltd., Wuhan, China). The digested DNA was purified using commercially available magnetic beads (MGI Tech Co., Ltd., Wuhan, China), and DNA libraries were prepared (after end repair, adapter ligation, and PCR amplification) using the DNA construction kit (MGI Tech Co., Ltd., Wuhan, China). The library was quantified using the Qubit 4 Fluorometer (Invitrogen); the ExKubit dsDNA Assay Kit and Agilent 2100 (Agilent Technologies, Santa Clara, CA) were used to control fragment length. DNA nanoballs (DNBs) were subsequently prepared from the library using rolling circle amplification (BGI Genomics, Wuhan, China). Next, a loading sample was prepared and sequenced on the BGISEQ-50 platform (BGI Genomics, Wuhan, China) using the 50–base read length (SE50) sequencing strategy. Hela cells and *Acinetobacter baumannii* (ATCC 19606) were used as negative and positive controls, respectively, and were consistently included in the analysis of each batch of samples. A specific synthetic sequence tagged onto each specimen was used as an internal standard to monitor the whole process. The quality of the extracted DNA, the sequencing library, DNBs, and final sequence data were all measured to a great degree of accuracy. The workflow of mNGS test was shown in **Figure 1B**.

For the bioinformatics aspect of mNGS, raw sequencing data were processed, and high-quality data were selected after removing adapter and low-quality sequences. The high-quality data were then mapped to the human genome (hg19; https://www.ncbi.nlm.nih.gov/assembly/GCF_000001405.13/) using the Burrows–Wheeler Alignment (BWA; http://bio-bwa.sourceforge.net/) method and stripped for annotated human genome data (Li and Durbin, 2009). The remaining sequencing data were simultaneously aligned against Pathogenic metagenomics database (PMDB) microbial genome databases, which included the genomes of viruses, bacteria, fungi, and parasites, using BWA, to generate the original mapping list. The PMDB database contained sequence data from 6,039 bacteria, 2,700 DNA viruses, 1,064 fungi, 234 parasites, and 137 mycoplasma/chlamydia (all associated with human disease). Reference genomes in the database were downloaded from the National Center for Biotechnology Information (ftp://ftp.ncbi.nlm.nih.gov/genomes/).

### Statistical analysis and data visualization

Bioinformatics analyses and data visualization were performed using the R software. The type of specimen, genome length, sequencing depth, and the throughput rate of the platform will affect the reads number in mNGS. We chose the reads per kilobase of transcript per million mapped reads (RPKM) as the normalization method for mNGS reads, based on the study by Liu et al. (Liu et al., 2022c) and the sequencing characteristics of this study (single-end sequencing). RPKM was calculated using the formula: gene reads/[the total mapped reads (millions) × genome length (KB)].

The Shapiro–Wilk test was used to determine whether the quantitative data conformed to a normal distribution. The Student’s t-test and Wilcoxon rank test were used to compare two groups that followed a normal or an abnormal distribution, respectively. The Pearson chi-squared (χ^2^) test or the Fisher’s exact test was used for the comparison of categorical data frequencies. The correlation between two different indicators was analyzed and expressed as Spearman’s r values. A receiver operating characteristic (ROC) curve was drawn for the selection of the best indicator of the true-positive specific pathogen. We applied the Youden index to determine the cutoff values for log(RPKM) and read counts in the ROC curve. The Youden index (*J*) is main summary statistic of the ROC curve used in the interpretation and evaluation of a biomarker, which defines the maximum potential effectiveness of a biomarker (Youden, 1950; Faraggi, 2000). A *P*-value of < 0.05 was used as a measure of significance.

## Results

### Clinical characteristics

The 310 participants who enrolled in our study were all hospital inpatients, comprising 96 women (31%) and 214 men (69%), with a median age of 64.5 [interquartile range (IQR), 50–74], range from 0.75 to 93 years. The median duration of hospitalization was 16 days (IQR, 11–23). Of the 310 participants, 152 were admitted to the intensive care unit (ICU), 79 died during therapy (equal to a 25% 30-day mortality rate), 79 received empiric antifungal drug therapy before mNGS, and 268 were immunocompromised. The causes of immunosuppression are classified in **Table 1**.

**Table 1:** Patient and sample characteristics.

Characteristics	Value
Patient demographics (n = 310)	
Age (years)	
Median (IQR)^1^	64.5 (50–74)
Range	0.75–93
Gender, n (%)	
Female	96 (31)
Male	214 (69)
Hospitalization, n (%)	
Patients, total	310 (100)
In hospital	310 (100)
In intensive care unit	152 (49)
Days hospitalized, median (IQR)	16 (11–23)
30-day mortality, n (%)	79 (25)
Immunocompromised, n (%)	268 (86.5)
On empiric antifungal drugs at time of body fluid collection, n (%)	79 (25)
Causes of Immunodeficiency, n (%)^2,3^	
Diabetes mellitus	21 (7.8)
Hypoproteinemia	45 (16.8)
Cancer	25 (9.3)
Hematologic disease	23 (8.6)
Renal disease	12 (4.5)
Gastrointestinal disease	14 (5.2)
Iatrogenesis^4^	28 (10.4)
Advanced age or organ failure	51 (19)
Surgery and trauma	18 (6.7)
Other^5^	31 (11.6)

^1^IQR, interquartile range. ^2^The percentage is based on all immunodeficient patients participating in the study. ^3^Because some patients suffer from a variety of diseases that may lead to immunodeficiency, only the primary disease or the disease contributing most to the immunosuppression was counted. ^4^Iatrogenic immunodeficiency mainly refers to the application of immunosuppressants such as corticosteroids, radiotherapy, or chemotherapy in patients with cancer (outlined in the “Cancer” category). ^5^The “Other” category includes the incomplete development of immune function in infants, immunosuppression induced by severe infection, etc.

### Identification of fungi by CTMs and mNGS

**Figure 2:**
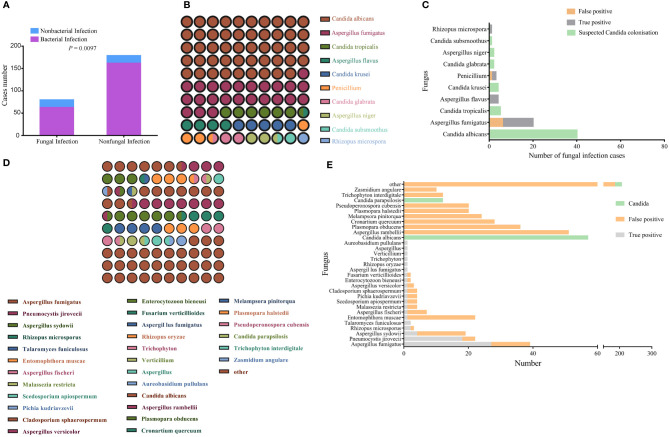
Identification of pathogenic fungi by conventional culture methods and mNGS. **(A)** Bacterial infection status of the fungal infection and non-fungal infection groups. The comparison between the two groups was made using the Pearson chi-squared (χ2) test. *P* < 0.05 indicates a significant difference. **(B)** Matrix of fungal species detected the culture method; the number of points represents species detection frequency. **(C)** Profile of fungal species detected by the culture method that distinguishes between colonization, false positives, and true positives. **(D)** Matrix of fungal species detected by mNGS; the number of points represents detection frequency (species with frequencies <10 were all grouped together). **(E)** Profile of fungal species detected by mNGS that distinguishes between colonization, false positives, and true positives; the number of points represents detection frequency (species with frequencies <10 were grouped other).

Of the 310 study participants, 80 patients were diagnosed with respiratory IFIs, of which 63 cases showed signs of bacterial co-infection. One hundred seventy-nine patients were excluded from further analyses of fungal infection; of these, 162 participants were diagnosed with bacterial infection, and 17 were diagnosed with non-infectious disease (**Figure 2A**). In addition, *Candida* spp. were detected in the BALF of 51 patients. Because pulmonary *Candida* infection is rarely confirmed by histopathology, the *Candida*-positive patients were placed into the suspected *Candida* colonization group.

In comparison with the non-fungal infection group, the percentage of immunosuppressed patients was higher in fungal infection group (**Table 2**). CMTs identified 82 fungal strains in 71 samples, of which the most common was *Candida albicans*, accounting for 48.7% of all strains (**Figure 2B**). Nineteen “true positive samples” were eventually selected, in which 22 strains of five species were detected. *Aspergillus fumigatus* was the most frequently detected species; 14 strains of *A. fumigatus* were detected (**Figure 2C**). In contrast, mNGS detected suspicious fungal sequences in 290 patients. One hundred forty fungal strains were detected 617 times; *C. albicans* was again the most common, accounting for 9.2% of all strains (**Figure 2D**). mNGS positively identified 51 patients who were clinically considered to have fungal infection, in which 20 kinds of species were detected 68 times. The most common species was *A. fumigatus*; 27 A*. fumigatus* strains were detected, accounting for 39.7% of all identified strains (**Figure 2E**).

**Table 2:** Demographic characteristics of study participants with fungal or non-fungal infections of the respiratory tract.

Group	Age (years)	Gender, n (%)		ICU, n (%)		Immunodeficiency, n(%)	
		Female	Male	Yes	No	Yes	No
Fungal infection	59.7 ± 18.6	29 (36.3)	51 (63.7)	42 (51.9)	38 (48.1)	80 (100)	0 (0)
Non-fungal infection	55.7 ± 21.4	53 (29.6)	126 (69.4)	72 (40.6)	107 (59.4)	146 (81.6)	33 (18.4)
P-value	0.062	0.288		0.06		<0.001	

P < 0.05 indicates a significant difference.

### Evaluation and optimization of mNGS-mediated diagnostic efficacy

**Figure 3:**
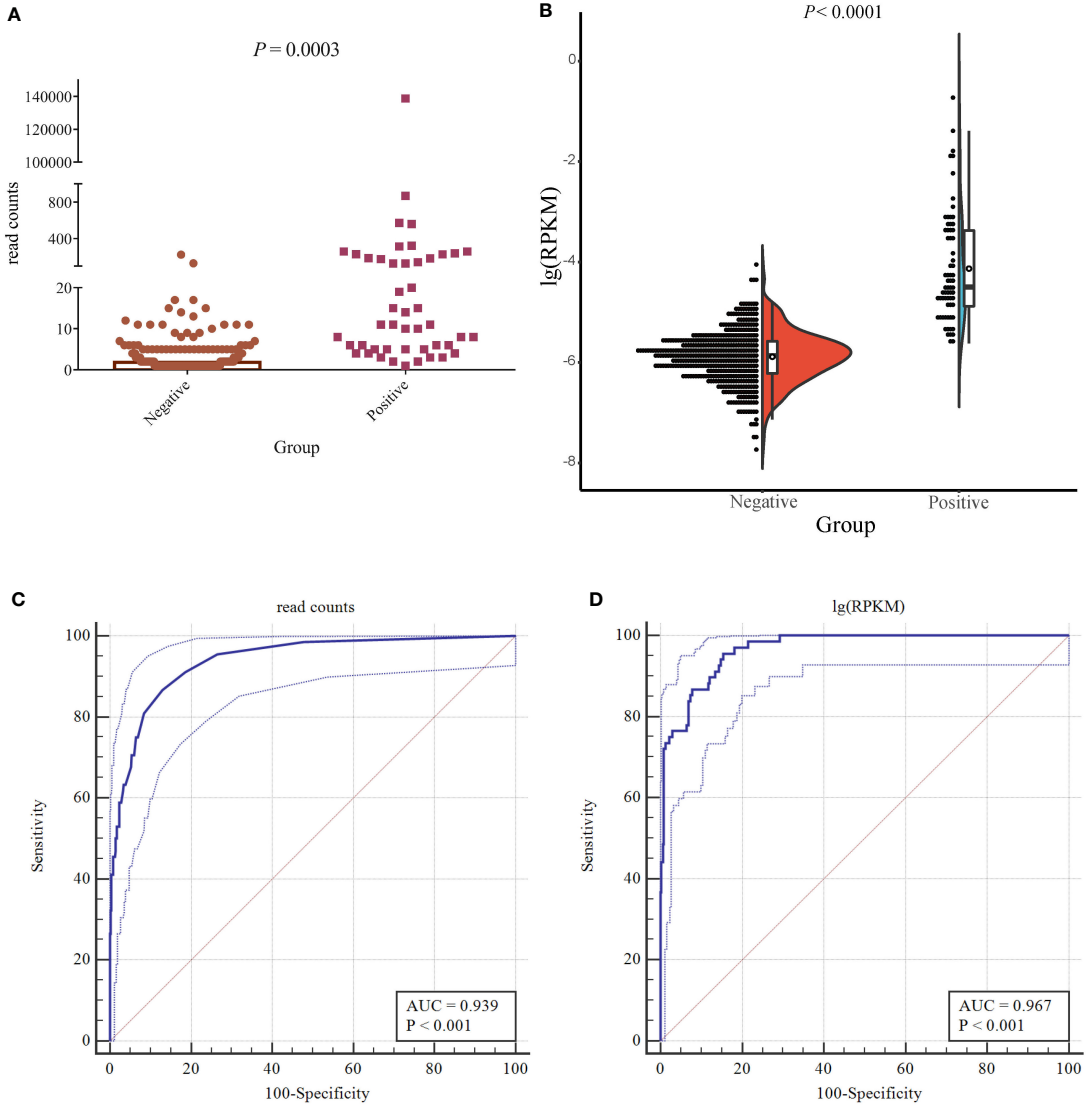
Accuracy of mNGS. **(A)** The difference in the number of detected reads between true- and false-positive fungi. **(B)** The half violin diagram shows the difference in lg(RPKM) values between true- and false-positive fungi. **(C, D)** Evaluating the performance of lg(RPKM) **(C)** and read counts **(D)** between the true- and false-positive pathogenic fungal groups using ROC curves.

The ROC curve was used to evaluate the performance of mNGS, whereas the final clinical diagnosis was used as the gold standard. In mNGS, the number of reads is affected by the type of specimen, genome length, sequencing depth, and the throughput rate of the platform. The use of read numbers for laboratory-based diagnosis may therefore lead to bias. To rule out these effects, the number of sequencing reads was replaced with the logarithm of reads per kilobase per million mapped reads [lg(RPKM)]; RPKM was calculated using the formula: gene reads/[the total mapped reads (millions) × genome length (KB)]. The results showed that read number and lg(RPKM) were significantly higher when infection was classed being “true positive” (**Figures 3A, B**). In terms of diagnostic efficacy, both read number and lg(RPKM) can effectively evaluate fungal infection. The area under the ROC curve (AUC) for the read number was 0.939, and the cutoff value calculated according to the Yoden index was greater than 4. Concurrently, we obtained a sensitivity rate of 86.76% and a specificity rate of 86.98% (**Figure 3C**). We found that the AUC value for lg(RPKM) was 0.967, and the cutoff value calculated according to the Yoden index was greater than −5.44. The sensitivity and specificity rates were 95.59% and 84.6%, respectively (**Figure 3D**). The DeLong method (DeLong et al., 1988) was used to compare the two ROC curves, showing that lg(RPKM) had significantly higher diagnostic efficiency in relation to fungal detection than the read count method (**Table 3**).

**Table 3:** Pairwise comparison of ROC curves.

Variable	lg(RPKM)	Reads
AUC	0.967	0.939
95% CI	0.948 to 0.981	0.915 to 0.958
Difference between areas		0.0279
Standard error		0.0125
z-statistic		2.234
Significance level		*P* = 0.0255*

*P < 0.05 indicates a significant difference.

### Comparison of the diagnostic efficiencies of mNGS and CMTs

**Figure 4:**
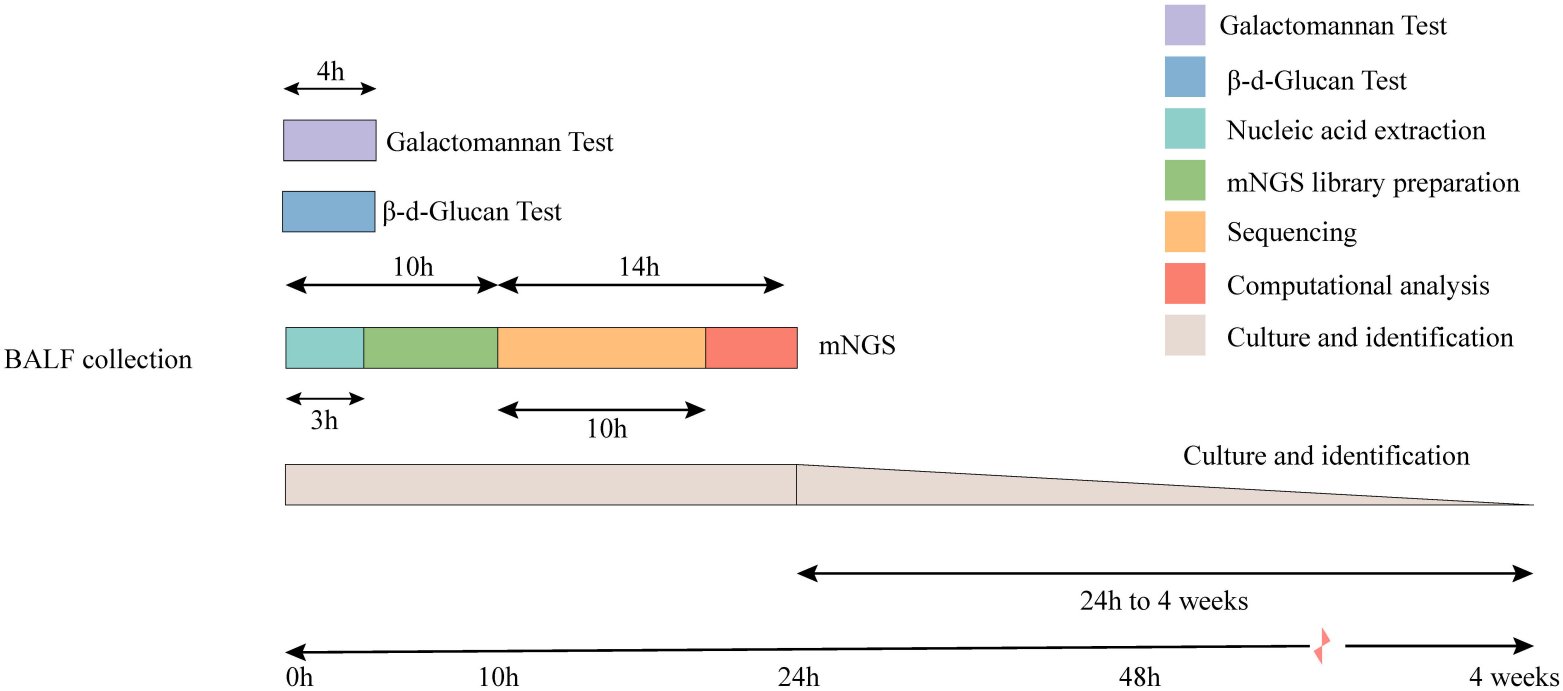
Timeline for mNGS and CMTs. Culture-based pathogen identification can take days to weeks. In comparison, mNGS testing using the BGI sequencing platforms has an overall turnaround time of 24 h.

**Figure 5:**
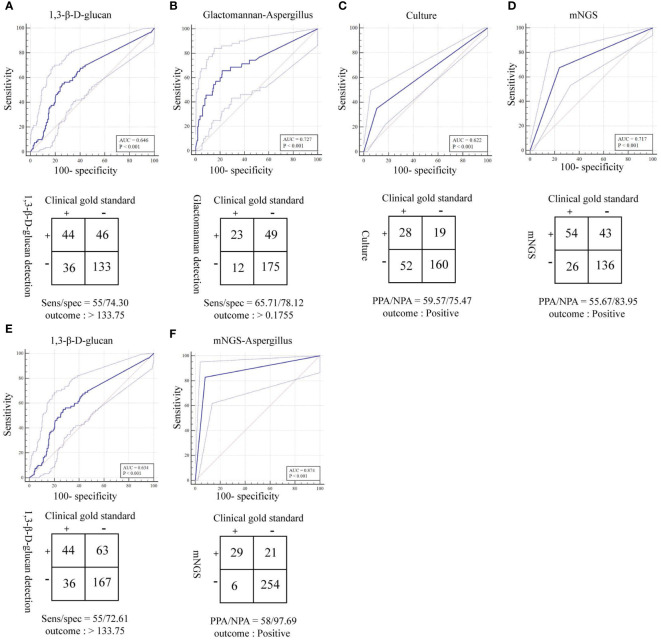
ROC curves and 2 × 2 contingency tables showing the respective diagnostic performance for different fungal detection methods; plotted are mNGS test sensitivities and specificities, relative to the clinical gold standard. PPA and NPA are shown in lieu of sensitivity (sens) and specificity (spec), respectively, if a composite standard was used. **(A)** Diagnostic efficacy of the 1,3-β-D glucan test (G test) for fungi (removal of patients with suspected *Candida* infection). **(B)** Diagnostic efficacy of the galactomannan test (GM test) for Aspergillus. **(C)** Diagnostic efficacy of the culture method for fungi. **(D)** Diagnostic efficacy of mNGS for fungi. **(E)** Diagnostic efficacy of the 1,3-β-D glucan test (G test) for fungi. **(F)** Diagnostic efficacy of mNGS for Aspergillus.

The total turnaround time (TAT) of mNGS (from nucleic acid extraction to result reporting) is ~24 h, which is higher than that of the G and GM tests. However, the TAT of mNGS was much lower than the average duration of fungal cultures (**Figure 4**). To clarify the actual diagnostic performance of mNGS in this study, the cutoff value of lg(RPKM) was used to identify the presence of IFI-causing fungi in the study subjects. After taking the final clinical decision as the gold standard, the results showed that the AUC for lg(RPKM) was higher than for the G and fungal culture tests, meaning that lg(RPKM) was superior at identifying fungal infections in patients. Moreover, the positive and negative coincidence rates were also higher than those of the culture method (**Figure 5A, C, D**). After eliminating the influence of *Candida* infection on the test results, the AUC value of the G test improved from 0.634 to 0.646 (**Figures 5A, E**). In identifying *Aspergillus* spp. infection, the AUC values of the GM assay and mNGS were 0.727 and 0.874, respectively. The diagnostic performance of mNGS was significantly better than that of GM test (*P* = 0.0104; **Figures 5B, F**).

### Correlation analysis between laboratory indices and fungal infection

**Figure 6:**
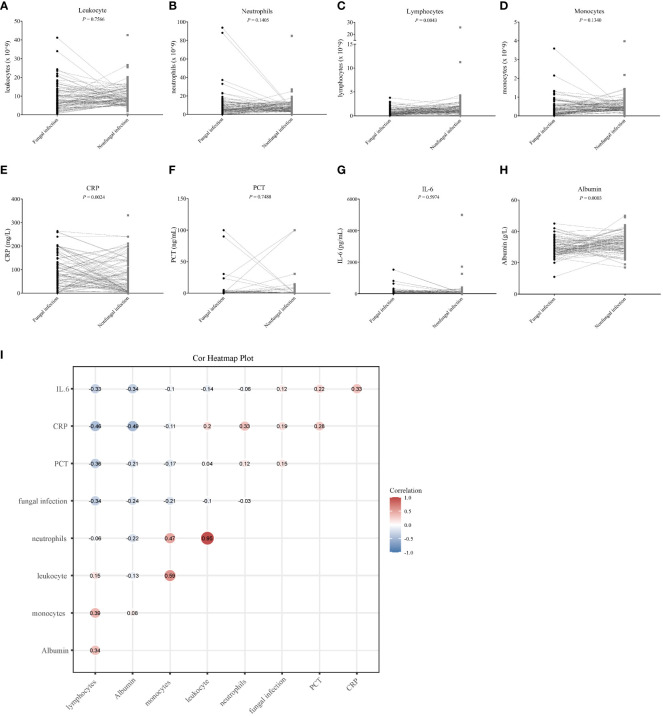
The correlation between eight clinical infection indicators and fungal infection. **(A–H)** Differential analysis of eight clinical infection indices in the fungal and non-fungal infection groups. The Student’s t-test was used to evaluate the significance of any differences detected, and values with a *P* < 0.05 were considered as significant. **(I)** Heat map corresponding to the correlation analysis between the clinical infection indicators and fungal infection; a correlation coefficient is shown at each spot of the matrix. The Spearman method was used for the correlation analysis.

Indicators such as white blood cell count, CRP, PCT, IL-6, and albumin may reflect the state of the body after infection to varying degrees. We used the Spearman method to analyze the correlation of eight indicators with the presence of fungal infection. The results showed that the serum albumin levels and lymphocyte counts were significantly lowered, and the CRP levels were significantly raised after fungal infection (**Figures 6C, E, H**). Leukocyte, neutrophil, monocyte, PCT and IL-6 were not significantly different between the fungal and non-fungal infection groups (**Figures 6A, B, D, F, G**). In addition, this study found that serum albumin as well as lymphocyte and monocyte counts were significantly negatively correlated with fungal infection, whereas the CRP and PCT values showed a significant positive correlation with fungal infection (**Figure 6I**; **Table 4**).

**Table 4:** The correlation between conventional indicators and fungal infection was analyzed using the Spearman’s method.

Conventionalinfection indicators	Spearman’srank correlation coefficient (rho)	Significance level	95% Confidence interval for rho
Albumin	−0.241	*P* = 0.0001*	−0.352 to −0.122
CRP	0.191	*P* = 0.0020*	0.0709 to 0.306
IL-6	0.119	*P* = 0.0558	−0.00295 to 0.237
Leukocyte count	−0.103	*P* = 0.0978	−0.222 to 0.0190
Lymphocyte count	−0.344	*P* < 0.0001*	−0.447 to −0.232
Monocyte count	−0.209	*P* = 0.0007*	−0.322 to −0.0891
Neutrophil count	−0.0292	*P* = 0.6403	−0.151 to 0.0930
PCT	0.147	*P* = 0.0175*	0.0261 to 0.265

*P < 0.05 indicates a significant correlation.

## Discussion

IFIs are prevalent in critically ill patients and individuals suffering from pulmonary disease. Furthermore, diagnosing IFIs is challenging because of the non-specific symptoms and a low diagnosis rate (Liu et al., 2022b). The diagnosis of IFIs in immunocompromised patients is even more difficult, as the symptoms are more insidious than those of patients without immunosuppression and the sensitivity of serological tests is lower following the inhibition of the immune response. In immunocompromised patients, the major instigators of fungal pulmonary infection are *Aspergillus* spp. and *Pneumocystis jirovecii* (Fishman and Rubin, 1998; Chong et al., 2006; Brown et al., 2012). Infection with fungi such as *Histoplasma* spp., *Blastomyces* spp., and *Coccidioides* spp. is epidemic in America and Africa but is rare in China, which was in accordance with our results (Azoulay et al., 2020).

In this study, all subjects were suspected of pulmonary IFIs and showed symptoms or radiographic signs of fungal infection, 53% of them were ultimately judged to have a simple bacterial infection. Thus, in the absence of a laboratory diagnosis, bacterial infections can interfere with fungal infections. The occurrence of fungal-bacterial co-infection may be related to the immunocompromised state of the subjects in the fungal group, which was generally immunocompromised and severe. The characteristics of infection in the case of bacterial–fungal co-infection, as well as the specific mechanisms of interaction, need to be further investigated.

*Candida* spp. are the most frequently detected fungal pathogen in BALF; the top five species are *Candida albicans*, *Candida glabrata*, *Candida tropicalis*, *Candida parapsilosis*, and *Candida krusei* (Williams et al., 2013). Invasive candidiasis usually refers to candidemia and deep-seated infections such as those resulting in intra-abdominal abscesses, peritonitis, or osteomyelitis (Pappas et al., 2018). Despite the ubiquitous existence of *Candida* spp. in respiratory specimens, especially in the critically ill and immunosuppressed patients, true histopathologically confirmed that *Candida* pneumonia is rare (Meersseman et al., 2009; Hamet et al., 2012).

Fungal culture is time-consuming, and the positive rate of growth can be influenced by potential initiation of empirical antibiotics therapy. In contrast, mNGS, which rely on sequencing the DNA already present in clinical samples, can be completed in a short time. In our study, mNGS detected more fungal species than CMTs and with higher degrees of sensitivity and specificity. However, the false-positive rate of mNGS was significantly higher than that of CMTs. Therefore, it is necessary to distinguish between true positives and false positives when interpreting mNGS results. We used lg(RPKM) and read number as indicators for plotting ROC curves and evaluating the performance of mNGS. According to our analysis, lg(RPKM) and read number all showed excellent capacity for IFI diagnostics, and an identification cutoff was also established. Among these prediction parameters, lg(RPKM) exhibited superior performance. To further clarify the actual diagnostic efficacy of the lg(RPKM) threshold in fungal detection, we should apply this threshold to the all cases of suspected fungal infection.

Detection of (1,3)-β-d-glucan (BDG; a cell wall component of all fungi except *Cyrptococcus* and *mucormycetes*) and GM (an *Aspergillus* spp. hyphal cell wall constituent) in the blood or BALF of patients is widely used for the diagnosis of IFIs. The BDG is an adjunct marker of IFIs, but it does not distinguish between *Candida* spp., *Aspergillus* spp., and *P*. *jirovecii*, which are all associated with a high false-positive rate (Theel and Doern, 2013). In our study, the false-positive rate of BDG in the diagnosis of IFIs was 58.87%, which was lowered to 51.11% on subtraction of the *Candida*-associated bias and infection with other suspected colonizing fungi. Our study indicates that the combination of mNGS and BDG detection can improve the diagnostic efficiency of IFIs. According to reports, GM specifically identifies aspergillosis (Donnelly et al., 2020), with 21%–86% sensitivity and 80%–92% specificity in serum samples and with 60%–100% sensitivity and 68%–100% specificity in the BALF (Miceli and Kauffman, 2017). In our study, the sensitivity rate of BALF GM in the diagnosis of *Aspergillus* infection was 65.71% and the specificity rate was 78.12%, which was consistent with previous studies (Miceli and Kauffman, 2017; Donnelly et al., 2020). The AUC value for mNGS in the diagnosis of *Aspergillus* infection was 84.7%, which was significantly higher than for GM (72.7%). These findings indicate that mNGS could be especially useful in the diagnosis of *Aspergillus* infection.

We found that the efficiency of *P*. *jirovecii* detection was much higher for mNGS than that for CMTs. *P*. *jirovecii* is an important opportunistic fungus that can colonize the respiratory tract and be transmit from person to person *via* the airborne route. Thus, iatrogenically immunocompromised patients are the principal risk group for *P. jirovecii* infection. It was reported that one-third of *Pneumocystis* pneumonia cases occur in HIV-negative immunocompromised patients, possibility of due to an increase in infection when the CD4+ lymphocyte counts drop below 200 cells/µl (Morris et al., 2004; Laura et al., 2016). Immunofluorescence staining and/or PCR of sputum (in a sputum induction test) or BALF samples are feasible methods for the diagnosis of *P. jirovecii* pneumonia (PJP). However, the positive rate of direct immunofluorescent staining is relatively low, and PCR requires prior knowledge of the organisms present in the sample (Alexandre et al., 2016). As a new pathogen detection strategy, mNGS reached a sensitivity rate of 100% in the diagnosis of PJP, according to a study of HIV-negative immunocompromised patients (Jiang et al., 2021). Jiang et al. found that the sensitivity of mNGS was remarkably higher than those of Gomori methenamine silver staining (GMS; 25.0%) or the serum BDG test (67.4%) (Jiang et al., 2021). The specificity of mNGS (96.3%) also significantly surpassed the serum BDG test (81.4%) (Jiang et al., 2021). Similar conclusions were reached by other independent studies, in which mNGS outperformed GMS and BDG detection techniques for *P. jirovecii* infection (Xu et al., 2021; Sun et al., 2022; Duan et al., 2022).

Most of the current studies on the diagnosis of IFD by mNGS are clinical case reports (Chen et al., 2021; Wang et al., 2022; Cui et al., 2022; Hu et al., 2022). Several studies have focused on the diagnosis of pulmonary infections by mNGS, Peng et al. reported on 60 patients with suspected pneumonia in severe immunodeficiency and analyzed the diagnostic efficacy of mNGS versus CMTs for pneumonia (Peng et al., 2021). Peng et al. concluded that mNGS was more effective than CMTs for viral infections in the lung, but the opposite was true for fungal infections. This differs from the conclusion that we reached. In the study by Peng et al., many *Aspergillus*-positive patients tested negative for mNGS because of the thick polysaccharide cell wall of *Aspergillus*, which made DNA extraction difficult. In our study, it has been possible to lyse *Aspergillus* and extract the relevant DNA for amplification detection well, benefiting from the development of pre-treatment techniques for mNGS. A related study by Lin et al., which included 69 immunodeficient patients with suspected pneumonia, concluded in agreement with us that the diagnostic efficacy of mNGS was significantly better than that of CMTs (Lin et al., 2022). Both studies by Peng et al. and Lin et al. compared the sensitivity and specificity of the two methods through simple statistical calculations, without using statistical tools such as ROC curves to comprehensively analyze the difference in diagnostic efficacy between mNGS and CMTs, and did not give diagnostic cutoff values. To the best of our knowledge, the current study is the largest study to evaluate the diagnostic performance of mNGS in immunocompromised patients with invasive fungal disease.

Our study has several limitations. First, the DNA extraction method that we used can be generically applied to viruses, bacteria, fungi, and parasites. Thus, the DNA extraction efficacy for fungi could have been suboptimal due to the thickness of the fungal cell walls, which may require more vigorous DNA extraction methods. Second, we could not definitively distinguish between *Candida* infection and colonization, so this aspect of the data was excluded in certain types of analysis. Finally, the mNGS includes indicators such as read counts, [lg(RPKM)] in addition to genome coverage, and the relative abundance of each organism. Because a single indicator can only reflect part of the characteristics of a test system (Yuan et al., 2019), it is necessary to use multiple indicators to evaluate the test performance of mNGS in combination.

In conclusion, we established mNGS to be a promising diagnostic technique for IFIs. Compared with CMTs, mNGS has multiple advantages in identifying true-positive fungal infections. Although many challenges remain in the clinical application of mNGS, our study had shed new light on the applicability of this novel methodology for the diagnosis of IFIs.

## Data availability statement

The datasets presented in this study can be found in online repositories. The name of the repository and accession number can be found below: CNGB Sequence Archive (CNSA) of China National GeneBank DataBase (CNGBdb); accession number CNP0003204.

## Ethics statement

The studies involving human participants were reviewed and approved by Medical Ethics Committee of Liaocheng People’s Hospital.

## Author contributions

CW, ZY, and FP designed and drafted the paper; JF, SC, DB, HZ, PS, and XY collected medical records; CW, WX, and XJ performed statistical analyses; CW analyzed the mNGS data; FP and QZ revised the article. All authors read and approved the final article. All authors contributed to the article and approved the submitted version.

## Funding

This research was supported by the Medical Science and Technology Development Foundation of Department of Health of Shandong Province, China (Grant No. 2019WS113).

## Conflict of interest

The authors declare that the research was conducted in the absence of any commercial or financial relationships that could be construed as a potential conflict of interest.

## Publisher’s note

All claims expressed in this article are solely those of the authors and do not necessarily represent those of their affiliated organizations, or those of the publisher, the editors and the reviewers. Any product that may be evaluated in this article, or claim that may be made by its manufacturer, is not guaranteed or endorsed by the publisher.
