# Supplementary material for: Application of metagenomic next-generation sequencing in the diagnosis of pulmonary invasive fungal disease
Source: Front Cell Infect Microbiol. 2022 Sep 27;12:949505. doi: 10.3389/fcimb.2022.949505 (PMC9551268; doi:10.3389/fcimb.2022.949505)
Supplement: Supplementary file 1 [file Table_1.docx]

**Table S1: IFD except for endemic mycoses diagnosis according to the revised criteria by the European Organization for Research and Treatment of Cancer/Invasive Fungal Infections Cooperative Group**

| **Proven** | Having a clinical picture consistent with IFD with one of the following  - Presence of histopathologic, cytopathologic, or direct microscopic examination consistent with yeast or a mold for a specimen that was collected from a sterile site  - Presence of positive culture from sterile sites  - Presence of positive blood culture |
| --- | --- |
| **Probable** | Presence all the following: one of host factors*, one of clinical criteria** AND one of microbiological criteria*** |
| **Possible** | Presence one of host factors and one of clinical criteria with lack of microbiological criteria |

*Host factors included history of neutropenia temporally related to the onset of fungal disease, receipt of an allogeneic stem cell transplant, prolonged use of corticosteroids, use of T cell immunosuppressants (i.e. cyclosporine and TNF-α blockers) or having inherited severe immunodeficiency.

**Clinical criteria included: lower respiratory tract fungal disease (supported by certain imaging features), tracheobronchitis, sinonasal infection, CNS infection or disseminated candidiasis.

***Mycological criteria included direct test (cytology, direct microscopy, or culture) or indirect testing (including galactomannan detection in aspergillosis or BGG detection in invasive fungal disease other than cryptococcosis and zygomycoses)
